# Altered retinal microRNA expression profile in a mouse model of retinitis pigmentosa

**DOI:** 10.1186/gb-2007-8-11-r248

**Published:** 2007-11-22

**Authors:** Carol J Loscher, Karsten Hokamp, Paul F Kenna, Alasdair C Ivens, Peter Humphries, Arpad Palfi, G Jane Farrar

**Affiliations:** 1 Smurfit Institute of Genetics, Trinity College Dublin, College Green, Dublin 2, Ireland; 2 Wellcome Trust Genome Campus, Sanger Institute, Hinxton, Cambridge, CB10 1SA, UK

## Abstract

**Background:**

The role played by microRNAs (miRs) as common regulators in physiologic processes such as development and various disease states was recently highlighted. Retinitis pigmentosa (RP) linked to *RHO *(which encodes rhodopsin) is the most frequent form of inherited retinal degeneration that leads to blindness, for which there are no current therapies. Little is known about the cellular mechanisms that connect mutations within *RHO *to eventual photoreceptor cell death by apoptosis.

**Results:**

Global miR expression profiling using miR microarray technology and quantitative real-time RT-PCR (qPCR) was performed in mouse retinas. RNA samples from retina of a mouse model of RP carrying a mutant Pro347Ser *RHO *transgene and from wild-type retina, brain and a whole-body representation (prepared by pooling total RNA from eight different mouse organs) exhibited notably different miR profiles. Expression of retina-specific and recently described retinal miRs was semi-quantitatively demonstrated in wild-type mouse retina. Alterations greater than twofold were found in the expression of nine miRs in Pro347Ser as compared with wild-type retina (*P *< 0.05). Expression of miR-1 and miR-133 decreased by more than 2.5-fold (*P *< 0.001), whereas expression of miR-96 and miR-183 increased by more than 3-fold (*P *< 0.001) in Pro347Ser retinas, as validated by qPCR. Potential retinal targets for these miRs were predicted *in silico*.

**Conclusion:**

This is the first miR microarray study to focus on evaluating altered miR expression in retinal disease. Additionally, novel retinal preference for miR-376a and miR-691 was identified. The results obtained contribute toward elucidating the function of miRs in normal and diseased retina. Modulation of expression of retinal miRs may represent a future therapeutic strategy for retinopathies such as RP.

## Abstract 2

MicroRNA expression profiling showed that the retina of mice carrying a rhodopsin mutation that leads to retinitis pigmentosa have notably different microRNA profiles from wildtype mice; further in silico analyses identified potential retinal targets for differentially regulated microRNAs.

## Background

MicroRNAs (miRs) are small noncoding RNAs that regulate gene expression at the post-transcriptional level in animals, plants, and viruses [1,2]. Mature miRs are produced in two steps after transcription of the primary miR transcript by RNA polymerase II [3]. Nuclear cleavage of the primary miR is mediated by Drosha and results in a short (about 75 nucleotides) hairpin precursor miR [3]. Following active transport to the cytoplasm by Ran and Exportin-5, the precursor miR is further processed by Dicer [4]. The end product is a mature miR (about 22 nucleotides) that, via incorporation into the RNA-induced silencing complex [5], appears to play crucial roles in eukaryotic gene regulation, primarily by post-transcriptional silencing. The effect of the mature miR depends largely on the level of base pairing with target sites, typically - but not exclusively - located on the 3' untranslated region of the mRNA [6,7]. Perfect or near perfect complementarity of the miR to the target usually results in cleavage of the mRNA [8,9], whereas imperfect base pairing leads to translational repression by various mechanisms, including stalling translation, altering mRNA stability or moving mRNAs into specific, translationally inactive cytoplasmic sites called 'P-bodies' [1,10]. Additionally, RNA-directed transcriptional silencing may guide interference at the nuclear DNA level by promoting heterochromatin formation [1,10,11].

Recently, the role played by miRs in various ubiquitous biologic processes, including developmental timing and patterning, left/right asymmetry, differentiation, proliferation morphogenesis, and apoptosis, was highlighted [1,12-15]. For example, in zebrafish embryo, intricate temporal and spatial expression patterns of miRs support a role for them in vertebrate development [16]. Aided significantly by progress in miR microarray technology, sets of miRs have been found to be highly or specifically expressed in various tissues, including brain, in physiologic states [17-19]. Similarly, specific patterns of miR expression profiles are emerging in disease states, such as various forms of cancer [20,21], cardiac hypertrophy [22], and polyQ/tau-induced neurodegeneration [23]. A comprehensive description of mammalian miR expression in different organ systems and cell types, including malignant cells but excluding the retina, was recently constructed based on small RNA library sequencing [24]. In relation to the eye, miR-7 has been shown to play an important role in photoreceptor differentiation in *Drosophila *[25] and other miRs, such as miR-9, miR-96, miR-124a, miR-181, miR-182, and miR-183, were found to be highly expressed during morphogenesis of the zebrafish eye [16]. In mouse, a number of miRs (for instance, miR-181a, miR-182, miR-183 and miR-184) were detected at high levels in various parts of the eye, including the lens, cornea, and retina [26,27]. Most recently, using microarray technology, 78 miRs were found to be expressed in retina, including 12 miRs, whose expression varied diurnally [28]. However, despite the accumulating data, little is known about the global miR expression profile of the mammalian retina in diseased states.

Retinitis pigmentosa (RP) is the most common form of inherited retinal degeneration, affecting more than one million individuals worldwide [29]. It is a debilitating eye disorder that is characterized by progressive photoreceptor cell death that eventually leads to blindness, for which no therapies are currently available [30]. The fundamental genetic causes for many forms of RP have been described; mutations in more than 40 genes have been linked to the disease [31]. Notably, mutations in the rhodopsin gene (*RHO*), which encodes a principal protein of photoreceptor outer segments, are responsible for approximately 25% of autosomal dominant forms of RP [29,32]. Experimental data from animal models of RP and human patients suggest that photoreceptors die prematurely by apoptosis [33,34]. However, much less is known about the chain of events that leads from the different mutations to eventual cell death, a process that can take decades in humans [35]. As mentioned above, altered miR expression is believed to play a crucial role in various diseases, including neuronal degeneration [23]. Similarly, altered miR expression may underlie some of the mechanisms that cause cellular dysfunction in RP, or indeed mechanisms that attempt to compensate for the disease phenotype; to date, however, there is no experimental evidence to support this hypothesis.

In the present study a miR expression profile in the mouse retina was generated using miR microarray technology and quantitative real-time RT-PCR (qPCR), and miRs with newly assigned retinal preference were identified. Given the emerging role of miRs in health and disease, the retinal miR expression profiles of a mouse model of RP carrying a mutant pro347ser *RHO *transgene (P347S) [36] and wild-type mice were compared. Notably, the results from the study provide the first evidence of modified miR expression profiles in retinal disease.

## Results

### MicroRNA expression profile in wild-type retina

**Figure 1:**
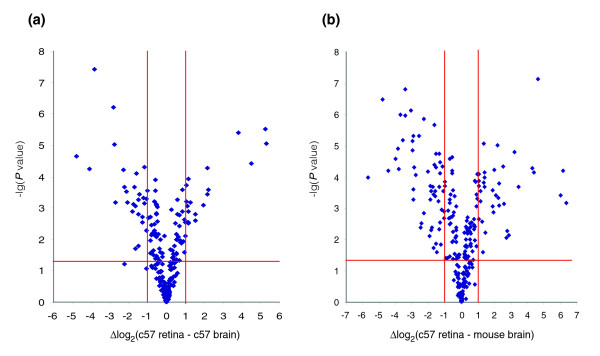
Volcano plots of miR expression in wild-type retina versus brain and mouse platform. Plots represent comparative miR expression profiles of **(a) **c57 retina versus c57 brain and **(b) **c57 retina versus mouse platform using Exiqon miR microarrays. X-axis indicate difference in expression level on a log_2 _scale, whereas the y-axis represents corresponding *P *values (Student's *t*-test) on a negative log scale; more lateral and higher points mean more extensive and statistically significant differences, respectively. Red lines indicate differences of ± 1, and significance level of *P *= 0.05. miR, microRNA.

Retinal miR expression was initially evaluated using microarray analyses. Comparison of the retina versus brain samples (Figure 1a) or the retina versus mouse platform samples (the latter prepared by pooling total RNA from eight different mouse organs; Figure 1b) resulted in large differences in miR expression profiles (Additional data file 1). Utilizing Exiqon microarrays (Exiqon, Vedbaek, Denmark), 104 out of 224 probes between the retina versus brain and 152 out of 222 probes between the retina versus mouse platform exhibited statistically significant (*P *< 0.05) differences in miR expression. More specifically, expression of 47 miRs in the retina versus brain and 81 miRs in the retina versus mouse platform changed by more than 2-fold (*P *< 0.05). In fact, the variance in relative expression was in excess of ± 6 on a log_2 _scale (Figure 1a,b). Note that Exiqon's microarray contains 488 mouse miR probes, but the probes that did not detect corresponding miRs in the above RNA samples were omitted from the plots; thus, the actual numbers of miRs included in Figure 1a and 1b were 222 and 224, respectively.

**Figure 2:**
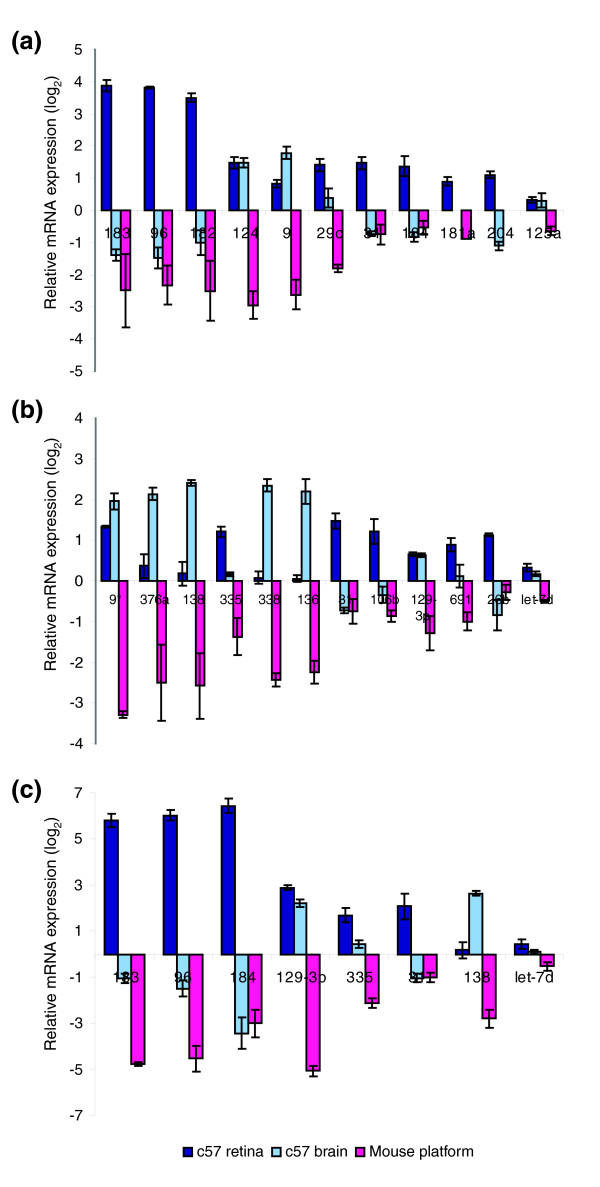
Comparative expression of selected miRs in the retina, brain, and mouse platform. Bars represent deviations from mean expression levels for each microRNA (miR) on a log_2 _scale in c57 retina (dark blue), c57 brain (light blue), and mouse platform (magenta). **(a) **Relative expression of some known retinal miRs. **(b) **Relative expression of miRs with novel retinal specificity. Panels a and b display data from miR microarray experiments. **(c) **Quantitative real-time reverse transcription polymerase chain reaction (qPCR) validation of expression of selected miRs. Note that columns are in descending order of difference between retinal and platform expression; y-axes are to different scales; and bars for miR-181a in brain and miR-204 in mouse platform are missing in panel a because of incomplete data.

Based on our miR microarray data, we undertook a semi-quantitative comparison of relative expression levels of some known retinal miRs (retinal specificity based on the work reported by Karali [26] and Ryan [27] and their colleagues) in retina, brain, and mouse platform (Figure 2a). Substantial variations in miR relative expression levels between retina and mouse platform were detected, ranging from a value of more than 6 (for miR-183 and miR-96) down to about 1 (miR-125a) on a log_2 _scale. Note, however, that these values are relative and therefore do not provide information about absolute miR levels. For example, miR-125a has a similar level of expression in retina, brain, and mouse platform, whereas miR-183 exhibits remarkable specificity for retina. Relative expression levels of additional miRs are given in Figure 2b, in a similar manner to those given in Figure 2a. Differences between relative miR expression levels in the retina versus mouse platform of up to 4 on a log_2 _scale were detected (Figure 2b). For example, miR-9*, miR-335, miR-31, miR-106b, miR-129-3p, miR-691, and miR-26b exhibited a relatively high level of expression in the retina when compared with the brain or the mouse platform. On the other hand, the relative levels of miR-376a, miR-138, miR-338 and miR-136 were high in the retina compared with the mouse platform, but even higher in the brain. Let-7d was used as a control to indicate ubiquitous miR expression in the retina, brain, and mouse platform (Figure 2b).

Selected miRs depicted in Figure 2a,b were chosen, and their relative expression levels quantified using qPCR in the retina, brain, and mouse platform (Figure 2c). Notably, a close correlation between qPCR and microarray data was found but, because of the sensitivity of PCR, data from qPCR analysis exhibited a higher dynamic range. For example, a difference in miR-183 expression between retina and platform samples was determined to be approximately 11 on a log_2 _scale by qPCR, as compared with about 6 on a log_2 _scale by microarray analysis. In case of miR-184 the disparity was more significant, with corresponding log_2 _values of approximately 9 (qPCR) versus 2 (microarray). Transformation of the qPCR log_2 _values into fold differences suggested that highly retinal specific miRs (for instance, miR-183 and miR-96) are expressed at more than a 1,000-fold greater degree in the retina than in the mouse platform. Recently described retinal miRs, such as miR-129-3p, also exhibited remarkable preference, with expressed being more than 250 times higher in the retina than in the mouse platform (Figure 2c).

**Figure 3:**
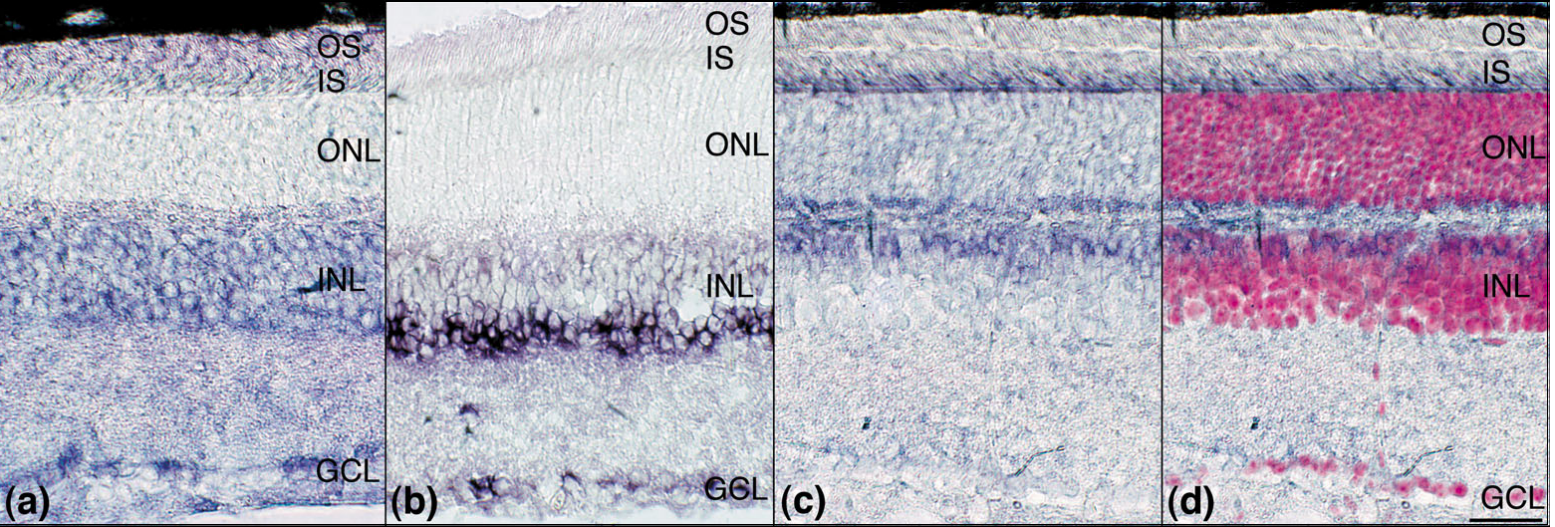
miR ISH analysis in the mouse retina. Eyes from 1-month-old c57 animals were fixed in 4% paraformaldehyde, and 12 μm cryosections were *in situ *hybridized with 5'-digoxigenin labeled locked nucleic acid (LNA) microRNA (miR) probes for **(a) **let-7, **(b) **miR-181a, and **(c,d) **miR-182. A false-colored (magenta) 4',6-diamidine-2-phenylindole-dihydrochloride (DAPI) nuclear staining is overlaid on the miR-182 *in situ *hybridization (ISH) label (panel d) to indicate the position of the nuclear layers. Scale bar: 25 μm. GCL, ganglion cell layer; INL, inner nuclear layer; IS, photoreceptor inner segments; ONL, outer nuclear layer; OS, photoreceptor outer segments.

Expressions of miR-1, miR-9*, miR-26b, miR-96, miR-129-3p, miR-133, miR-138, miR-181a, miR-182, miR-335 and let7-d were explored by *in situ *hybridization (ISH) using locked nucleic acid (LNA) probes (Exiqon). It is notable that only the analysis of let-7, miR-181a, and miR-182 produced detectable signals (Figure 3). Let-7 was expressed uniformly in the inner nuclear layer (INL) and labeling was also apparent in the ganglion cell layer (Figure 3a). MiR-181a was strongest in expression among these three miRs and was detected in the inner part of the INL, probably corresponding to amacrine cells and in the ganglion cell layer (Figure 3b). MiR-182 was expressed in the photoreceptor cells in the outer nuclear layer (ONL, Figure 3c). Both let-7 and miR-181a were mainly localized in the nuclear layers (Figure 3a,b), in contrast, miR-182 labeling was weaker in the ONL (cell bodies) but was strongly localized in the photoreceptor inner segments and between the ONL and INL, possibly in photoreceptor synapses (Figure 3c,d). Additionally, miR-182 labeling was also observed in the outer part of the INL. Labeling patterns depicted by ISH indicate cell type specific expression and possible differential intracellular targeting of these miRs, namely to the cell body or, in case of photoreceptor cells, to the photoreceptor inner segments and synapse.

### Altered miR expression in P347S retina

**Figure 4:**
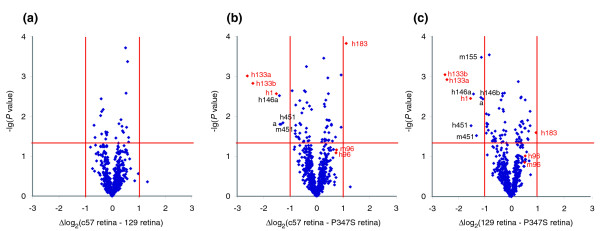
Volcano plots of miR expression in P347S and wild-type retinas. Plots represent comparative microRNA (miR) expression profiles of **(a) **c57 versus 129 retinas, **(b) **c57 versus P347S (mutant pro347ser *RHO *transgene) retinas, and **(c) **129 versus P347S retinas using Ambion miR microarrays. X-axis indicate difference of expression level on a log_2 _scale, while y-axis represents corresponding *P *values (Student's *t*-test) on a negative log scale; more lateral and higher points mean more extensive and statistically significant differences, respectively. Red lines indicate differences of ± 1 and significance level of *P *= 0.05. Labels are given for miRs with changes of higher than ± 1 (*P *< 0.05). MiR-1, miR-96, miR-133, and miR-183 are highlighted in red; h and m in labels refer to human and mouse miRs.

**Figure 5:**
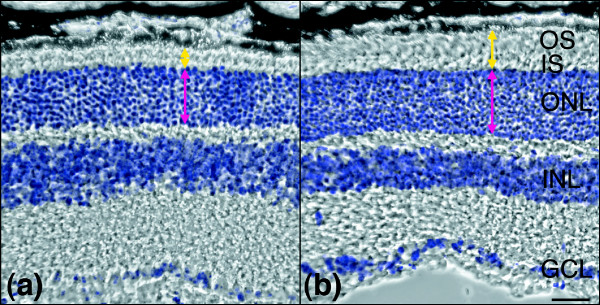
Comparative histology of 1-month-old c57 and P347S retinas. Eyes from 1-month-old c57 and P347S (mutant pro347ser *RHO *transgene) animals were fixed in 4% paraformaldehyde, 12 μm cryosections cut, and nuclei counterstained with 4',6-diamidine-2-phenylindole-dihydrochloride (DAPI). Phase contrast and fluorescent dark field (DAPI, false colored) microscopic images were overlaid to display histology of **(a) **P347S and **(b) **c57 retinas. Combined thicknesses of photoreceptor outer an inner segments (yellow arrows) and outer nuclear layer (magenta arrows) are indicated. Scale bar: 25 μm. GCL, ganglion cell layer; INL, inner nuclear layer; IS, photoreceptor inner segments; ONL, outer nuclear layer; OS, photoreceptor outer segments.

Given the emerging roles played by miRs in various diseases, we hypothesized that perturbed miR expression might contribute to some of the cellular events that underlie the pathology observed in RP. To seek experimental evidence to support this theory, miR expression profiles in retinas from an RP transgenic mouse model (P347S) [36] and c57 and 129 wild-type mice were compared by microarray analyses (Figure 4a,b,c and Additional data files 1 and 2). To reflect the adult miR expression pattern and to allow valid comparison of retinas from P347S and wild-type mice (the former with a progressive retinal degeneration and associated photoreceptor cell loss [36]), animals at age 1 month were chosen for the study. Figure 5 illustrates representative retinal histology of P347S (Figure 5a) versus wild-type c57 mice (Figure 5b) at 1 month of age. Compromised photoreceptor outer segments and a slightly decreased thickness of ONL (by ≤25%) were apparent in P347S mice (Figure 4a) when compared with wild-type control animals (Figure 5b). As a result, alterations in the retinal miR profile should be similar in magnitude to that of photoreceptor cell loss (approximately ± 25%). In contrast, larger changes in intracellular miR levels should reflect changes that have occurred because of altered regulation of miR expression in the P347S mutant retina. A 2-fold change threshold was set (+100% and -50%) to screen for miRs that differed in expression between P347S and wild-type mice.

In order to account for the mixed c57/129 genetic background of P347S mice, miR expression profiles in the retinas of P347S mice were compared with those in both c57 (Figure 4b) and 129 wild-type mice (Figure 4c); additionally, miR expression profiles of wild-type c57 versus 129 strains were directly compared (Figure 4a and Additional data file 2). In the c57 versus 129 comparison, minor variations in miR expression profiles were detected; out of 640 probes on the Ambion microarray, 25 gave significant (*P *< 0.05) but lower than 2-fold deviations between the two strains (Figure 4a). In contrast, the P347S versus c57 retina (Figure 4b) and the P347S versus 129 retina (Figure 4c) plots demonstrated marked alterations between the P347S and wild-type mouse miR profiles. Figure 4 parts b and c are almost identical and reveal statistically significant (*P *< 0.05) changes of 63 and 75 out of 640 miRs respectively, with only eight and nine miRs exhibiting greater than 2-fold (*P *< 0.05) changes between the P347S and wild-type c57 or 129 mouse retinal miR expression profiles. Using Exiqon LNA microarray technology, 16 probes had greater than 2-fold alterations (*P *< 0.05) between the P347S and c57 miR profiles (Additional data file 1). Note that for a number of miRs (for example, miR-1, miR-133, and miR-96), both Ambion and Exiqon microarrays detected similar alterations in expression between the P347S mutant and wild-type retinas.

**Figure 6:**
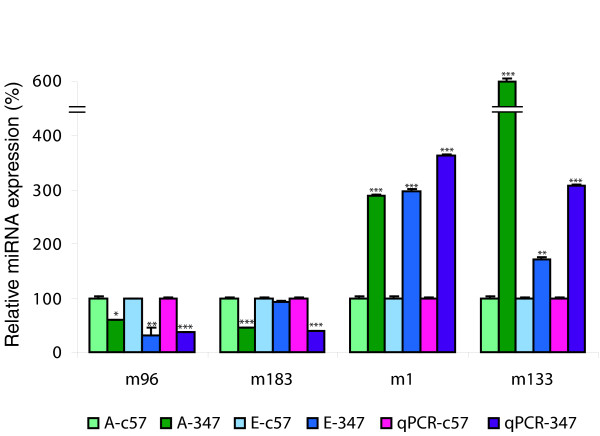
Differentially expressed miRs between c57 versus P347S retinas. Expressions of mouse microRNA (miR)-96, miR-183, miR-133 and miR-1 were analyzed using Ambion miR microarrays (green, 'A-' in legend), Exiqon miR microarrays (blue, 'E-' in legend), and quantitative real-time reverse transcription polymerase chain reaction (qPCR; magenta). Expression levels of each miR in P347S (mutant pro347ser *RHO *transgene; dark green, dark blue, and purple columns) versus c57 retinas (taken as 100%; light green, light blue and magenta columns) were compared. Note that the y-axis is discontinuous. *P < 0.05, **P < 0.01, and ***P < 0.001.

For qPCR validation, miRs with greater than 2-fold differences (*P *< 0.05) in expression between the P347S and wild-type mice were selected. Further criteria were that their signal values were above background for all samples and replicates, and probes corresponded to valid entries in the Sanger miR Database [37,38]. The above conditions were met by miR-1, miR-96, miR-133, and miR-183 (highlighted in red in Figure 4b,c); these miRs were therefore selected for qPCR quantification. Note, that in case of miR-96 greater than a 2-fold difference (*P *< 0.05) between the P347S and c57 mice was obtained with Exiqon microarrays only (Additional data file 1), while values from Ambion microarray analysis fell just below threshold. Some probes with greater than 2-fold changes (*P *< 0.05) represented unspecified Ambion or Exiqon miR sequences and thus were excluded from qPCR validation. Figure 6 displays corresponding data from the two different microarrays and qPCR analyses for miR-96, miR-183, miR-1, and miR-133. In general, a good correlation among data from qPCR and the two microarrays was found, with the exception of miR-183, for which the Exiqon microarray did not pick up the differential expression between mutant and wild-type retinas that was observed by qPCR (Figure 6). In summary, expression of miR-96 and miR-183 decreased by more than 2.5-fold (*P *< 0.001) in mutant retinas, whereas miR-1 and miR-133 increased by more than 3-fold (*P *< 0.001), as measured using qPCR. These results provide the first evidence for an altered miR expression profile in retinal disease.

Potential target transcripts for miR-96, miR-183, miR-1 and miR-133 predicted by miRanda [39] were retrieved from the Sanger miR Database [37]. In order to select for targets expressed in the retina, the transcripts were screened against seven Unigene mouse retina libraries and three gene lists derived from NEIBank [40] and serial analysis of gene expression (SAGE) studies in the mouse retina [41,42]. Matches based on gene names were extracted, resulting in a final subset of 1,664 miRanda predicted transcripts that are associated with known genes and are present in at least one retinal library or gene list (Table 1). The resulting miR targets were sorted by miRanda score, *P *orthologous group value, presence in the seven retinal libraries and three eye related lists (a score of 1 to 10), and predicted miR target sites per transcript (1 to 3). Additional data file 3 lists potential retinal target transcripts with the highest rankings for miR-96, miR-183, miR-1, and miR-133. Notably, transcripts of retinal disease genes, such as *Crb1 *(encoding Crumbs homolog 1), *Abca4 *(subfamily-D ATP-binding cassette member 4), *Pde6a *(phosphodiesterase 6A), *Prpf8 *(pre-mRNA processing factor 8) and *Prpf31 *(pre-mRNA processing factor 31 homolog), together with an additional 48 eye disease genes, are predicted to be targeted by these miRs (Additional data file 3). A subset of highly ranked potential targets for miR-96, miR-183, miR-1 and miR-133 are implicated in the visual cycle (for example *Abca4*, *Pitpnm1 *[membrane associated phosphatidylinositol 1], and *Pde6a*), in cytoskeletal polarization (for example, *Crb1 *and *Clasp2 *[CLIP associating protein 2]), and in transmembrane and intracellular signaling (for example, *Clcn3 *[chloride channel 3], *Grina *[*N*-methyl-D-aspartate-associated glutamate receptor protein 1], *Gnb1 *[guanine nucleotide binding protein beta 1 polypeptide] and *Gnb2 *[guanine nucleotide binding protein beta 2 polypeptide]). Notably, predicted targets of miR-96 and miR-183 also include apoptosis regulators, such as *Pdcd6 *(programmed cell death 6) and *Psen2 *(presenilin 2) and transcription factors (for example, *Asb6 *[ankyrin repeat and SOCS box-containing protein 6] and *Ndn *[Necdin]). Additionally, target transcripts for miR-1 and miR-133 comprise mRNA processing factors (for example, *Syf11 *[SYF2 homolog RNA splicing factor], *Prpf8*, and *Hnrpl *[heterogeneous nuclear ribonucleoprotein L]), an apoptosis inhibitor (*Faim *[Fas apoptotic inhibitory molecule]), and proteins that are involved in intracellular trafficking and motility (for example, *Ktn1 *[Kinectin 1]), *Actr10 *[ARP10 actin related protein 10 homolog], and *Myh9 *[non-muscle myosin heavy chain polypeptide 9]; see Additional data file 3).

**Table 1:** Overview of retinal miR target hits predicted by miRanda

Label	miRanda total hits	miRanda target genes	miRanda targets present in retinal libraries and lists
miR-96	994	857	518
miR-183	1064	902	541
miR-1	921	760	446
miR-133	1,097	925	592

miR, microRNA.

In summary, it has been demonstrated that miR expression in retinas from two wild-type mouse strains are very similar, and in contrast different patterns of expression between the retina, brain, and mouse platform were determined by miR microarray profiling. The results of the study suggest that the relative magnitude in expression of widely accepted retinal miRs varies remarkably in retina. Furthermore, the preferential expression in the retina of additional miRs, such as miR-376a and miR-691, represents a novel discovery. Retinal ISH analysis suggested cell type specific and intracellularly localized expression for the detected miRs. A comparative analysis between P347S and wild-type mouse retinas revealed a significant alteration in miR expression profiles in mutant mice, as evaluated by microarray analysis and validated by qPCR. More specifically, significant differences in expression of miR-1, miR-96, miR-133, and miR-183 in retina were observed between *RHO *mutant and wild-type mice. Potential retinal target transcripts for these miRs included, among others, genes implicated in retinal diseases and genes encoding components that are involved in apoptosis and intracellular trafficking.

## Discussion

A global expression profile of miRs currently available on microarrays was determined in mouse retina using two different microarray chemistries. Additionally, retinal preference/specificity was determined for miR-9*, miR-335, miR-31, miR-106, miR-129-3p, miR-691 and miR-26b by microarray analysis, and expression levels of miR-129-3p, miR-335 and miR-31 were also validated using qPCR. During the review process for this manuscript, Xu and coworkers [28] also reported retinal expression for some of these miRNAs. Little is known about the expression pattern, targets, or roles of these miRNAs. MiR-9* has previously been described as miR-131 [18], but it appears to be the sense strand of all three miR-9 predicted stem-loops. MiR-335 has been shown to be expressed in lung [43], miR-31 in colon [20], and miR-106 in megakaryocytes [44]. MiR-26b expression has been detected in mouse cortex and cerebellum [18], and more recently in embryonic stem cells [45], neuronal cells [46], and pancreatic cells [47]. MiR-129-3p was first cloned using a mouse pancreatic beta-cell line [47], whereas miR-691 was cloned from mouse embryo [48]. The roles of these miRs in the various tissues where they were originally isolated, or in retina, are largely unknown. Preferential expression in the retina was also observed for miR-376a, miR-138, miR-338, and miR-136 as compared with the mouse platform; it is notable, however, that these miRs are expressed at higher levels in brain than in retina. Indeed miR-136, miR-138, and miR-338 were previously cloned from the hippocampus and cerebral cortex [19].

Previously, miR-9, miR-29c, miR-96, miR-124a, miR-181a, miR-182, miR-183, and miR-204 were localized in the mouse retina by ISH [26-28]. However, ISH detection of other retina-specific miRs, including miR-213, miR-216, and miR-217, was unsuccessful in retina [26,27]. Among the 11 ISH probes investigated in the current study, only three (let-7, miR-181a, and miR-182) resulted in positive labeling in retina. Nevertheless, these three miRs exhibited an intricate pattern of expression, suggesting marked cell type specificity and also differential intracellular targeting. The most probable reason for the unsuccessful ISH detection of the other miRs tested is lower expression in terms of absolute quantities; other factors, such as secondary structure of probe or target, might also have contributed. Regarding photoreceptor specific expression, miR-182 has been shown to be strongly and exclusively expressed in rod photoreceptors [26], although the results of the present study also indicate labeling in the outermost part of the INL. This is in accordance with recent findings reported by Xu and coworkers [28], who demonstrated that expression of miR-96, miR-182, and miR-183 was not exclusive to photoreceptor cells in 4-month-old retinal degenerative 1 mice (rd1 [49]). Additionally, mir-124a expression is strong in photoreceptor outer segments and inner segment in adult mouse retina [26]. Marked retinal specificity of these miRs was verified by the microarray and qPCR analyses undertaken in the present study. The results obtained also indicate that although miR-124 and miR-9* are highly expressed in the retina as compared with the mouse platform, they are also expressed in the brain at a similar level. In fact, miR-124 and miR-9* are also known to be brain specific miRs [19,50,51].

In order to gain better insight into the possible association between miRs expression and retinal degeneration in diseases such as RP, retinal miR expression profiles of P347S versus wild-type mice were compared. Among others, expression of miR-96, miR-183, miR-1, and miR-133 exhibited significant alterations in P347S mice by microarray analysis, and these changes were validated by qPCR. The expression of miR-96 and miR-183 was reduced by more than 2.5-fold in P347S retinas compared with wild-type mouse retinas. The similar alteration in expression levels of these miRs may potentially be due to their close linkage (within 4 kilobases) on mouse chromosome 6qA3, thereby indicating that they may be co-regulated [38]. Indeed, recent studies in retina [28,42], inner ear [52], and dorsal root ganglia [53] suggest that miR-183, miR-96 and miR-182 may represent a conserved sensory organ-specific cluster of miRs, and that these miRs may potentially be under similar transcriptional control. In contrast, miR-1 and miR-133 levels increased by more than 3-fold in retinas of P347S mice. These miRs are also likely to be co-regulated [31] and have been described in relation to cardiac disease [22] and skeletal muscle proliferation and differentiation [54]. Interestingly, expression of miR-1 and miR-133 were found to be decreased in cardiac hypertrophy, whereas their over-expression inhibited hallmarks of induced cardiac hypertrophy *in vitro *and *in vivo *[22]. Similarly, the observed increased expression of miR-1 and miR-133 in the P347S retina may possibly suggest that a compensatory mechanism has been activated in the mutant retina in an attempt to prevent photoreceptor cell death.

Using a bioinformatics approach, potential target genes for miR-96, miR-183, miR-1, and miR-133 were predicted and screened against genes expressed in the mouse retina [41,42] and 488 genes linked with eye diseases [40]. The top 50 candidate target transcripts corresponded to genes that are, among others, involved in the visual cycle and transmembrane and intracellular signaling, and a number of retinal disease genes. Because expression of miR-96 and miR-183 is decreased, corresponding targets may potentially be upregulated in P347S mice. Notably, apoptosis and transcription factor genes are among the predicted targets for miR-96 and miR-183. In contrast, as miR-1 and miR-133 are upregulated, expression of their targets may possibly be suppressed in P347S mice. Many genes encoding factors that are involved in mRNA processing and splicing, and RNA-binding proteins belong to the predicted targets for miR-1 and miR-133. Additionally, genes encoding cytoskeletal and intracellular transport proteins, as well as an apoptosis inhibitor, were also predicted to be targets for these two miRs. These findings are in accordance with the suggestion that defective vectorial transport of rhodopsin in photoreceptor cells may be a possible precursor to cell death in P347S mice [36]. Potential activation of apoptosis genes and suppression of an apoptosis inhibitor is also in good agreement with the apoptotic death of photoreceptor cells observed in P347S retina, indeed emphasizing the role played by miRs in apoptosis [15]. MiR target transcript predictions, such as those made in the present study, are useful in highlighting the possible miR-dependent regulatory mechanisms that underlie retinal degeneration in P347S mice. However, further studies and experimental evidence is required to validate the predicted miR target transcripts.

Not all miRs with greater than 2-fold changes in expression between P347S and wild-type mice were followed up for qPCR validation. Unspecified Ambion and Exiqon company sequences, which are not as yet entered into the Sanger miR Database [37], were excluded from analysis but are listed in Additional data files 1 and 2. Other miRs, such as miR-451 and miR-146a (from Ambion microarray data) or miR-21, miR-23 and miR-140 (from Exiqon microarray data) were also left out from further analysis because these miRs exhibited very low levels of expression in the retina compared with the mouse platform. It was deemed that low signal-to-background ratios might have interfered with detection of the genuine expression levels for these miRs. The screening criterion implemented in the study (the threshold of at least a 2-fold change between P347S and wild-type mouse retinas) was chosen arbitrarily. It is notable that expression of more than 50 miRs changed significantly but by less than 2-fold (Figure 4 and Additional data files 1 and 2). Many of these may represent miRs whose intracellular expression might also have genuinely been altered in P347S retinas.

In the present study, the P347S transgenic model was selected for two reasons. *RHO*-linked RP is one of the most common types of RP, representing approximately 25% of all autosomal dominantly inherited RP cases in human patients [32]. In principle, the P347S transgenic animal model therefore potentially mirrors cellular events of a very frequent form of human RP. In addition, P347S mice are very useful because the retinal degeneration in this mouse model is relatively slow [36] compared with that in other *RHO*-linked transgenic RP lines, such as the Pro23His *RHO *mouse [55]. Slow degeneration in P347S mice provides a reasonable time frame for the mutant retina to develop into adulthood while maintaining a relatively normal histological structure and function, the latter demonstrated by normal electroretinography [36]. In particular, a time point of 1 month of age was chosen for the analysis because at this age P347S mice have a fully differentiated retina; in addition, although P347S mice carry a *RHO*-linked RP mutation with corresponding cellular dysfunctions, these mice exhibit only a minor decrease in photoreceptor cell numbers.

Note that in the present study a somewhat more significant degeneration in the P347S animals was detected compared with the original findings [36], which indicated little or no photoreceptor cell loss at this age. Regarding potential alterations in expression of individual miRs due to the above changes in cell composition in the P347S retina, they should in principle mirror the percentage of photoreceptor cell loss (approximately ± 25%). In light of this, it is unlikely that the significant changes observed in the expression of miR-96, miR-183, miR-1 and miR-133 are due to the altered cellular composition of the P347S retina. In contrast, Xu and coworkers [28] used rd1 mice with severe retinal degeneration to demonstrate retinal expression of miR-96, miR-182, and miR-183 in cells other than photoreceptor cells. In this case, altered expression of these miRs between wild-type and mutant retina was observed most likely because of the significant shift in cellular constituents (complete loss of photoreceptors) in the rd1 retina. It is also worth noting that the P347S mice are on a c57/129 mixed genetic background. The almost identical miR profiles between c57 versus 129 mice and the similar profiles between c57 versus P347S mice and 129 versus P347S mice support the view that the differences observed in retinal miR expression profiles, between P347S and wild-type mice, are a function of the presence of the *RHO *mutation in P347S mice and are not due to differences in genetic background.

## Conclusion

Data from this study combined with previous results demonstrate a widespread and intricate expression of miRs in the wild-type mouse retina. A small subset of miRs exhibits a high degree of tissue specificity, whereas others appear to be more ubiquitously expressed; there is a particular overlap between miRs expressed to relatively high degrees in retina and brain. Notably, potential function of miRs in retinal disease is highlighted by the first demonstration of an altered miR expression profile in retinal degeneration. Using a transgenic mouse model of a common form of human RP, widespread changes in miR expression profile were detected. In particular, the expression of two retinal specific miRs decreased significantly, whereas two non-retina-specific miRs, with a known role in muscle differentiation, proliferation and disease, increased extensively. Data presented in this study also contribute toward our understanding of the role played by miRs in the mouse retina by comparative miR expression profiling. From this analysis, a number of miRs were highlighted with newly identified retinal preference. At present, knowledge of the function of miRs in development, normal physiology, or disease states of the retina is limited. Notably, results from this study suggest that in *RHO*-linked RP the miR expression profile has been altered, mirroring observations in other disease states. Further studies should reveal the network of corresponding cellular targets and underlying mechanisms. Identifying disease-related miRs in RP models may provide a better understanding of the pathophysiology of retinal degeneration. Additionally, modulation of the expression of key miRs may potentially open future avenues for therapeutic development for retinopathies such as RP, in which - despite significant effort - there are currently no therapies.

## Materials and methods

### Experimental animals and RNA isolation

Transgenic P347S [36] and wild-type 129 and c57 mouse strains were used in these experiments. P347S animals are on a mixed c57/129 genetic background and carry a Pro347Ser mutation in the carboxyl terminal of *RHO*; this mutation has been identified in some autosomal dominant RP families [32]. To compensate for the extra *RHO *transgene, these mice were maintained on a mouse rhodopsin +/- background (*Rho*^+/-^) [30], resulting in a P347^+/-^Rho^+/- ^genotype. Retinal degeneration in these animals is slower than in most other RP models, with little or no photoreceptor cell loss at age 1 month and 50% of photoreceptors remaining at 4 to 5 months of age [36]. The spatial expression of *RHO *is normal and electroretinography amplitudes are comparable to that in the wild-type animals at 1 month of age [36]. Mice were maintained under specific pathogen free housing conditions. Animal welfare complied with the Association for Research in Vision and Ophthalmology statement for the Use of Animals in Ophthalmic and Vision Research and the European Communities Regulations 2002 and 2005 (Cruelty to Animals Act). At 1 month of age mice were killed by carbon dioxide asphyxiation.

For *in situ *hybridization studies, eyes from four animals from each strain were dissected and fixed in 4% paraformaldehyde for 4 hours at 4°C. For total RNA isolation retinas and brains were dissected immediately and extracted using the mirVana™ RNA Isolation kit (Ambion Inc., Austin, TX, USA), in accordance with the manufacturer's procedure. Tissue samples for total RNA were obtained in triplicate. In each sample six retinas were pooled, whereas individual brains were frozen in liquid nitrogen and homogenized over dry ice; 50 to 100 μg of the resulting powder was used for extraction. In order to represent the mouse body, a mouse total RNA platform was prepared by pooling total RNA from eight different mouse organs (liver, thymus, heart, lung, spleen, testicle, ovary, and kidney) from the Mouse Assorted Total RNA kit (Ambion Inc.).

### Microarray experiments

Two different miR microarray technologies (mirVana™ miRNA Bioarray [Ambion Inc.] and miRCURY™ LNA miR Array [Exiqon, Vedbaek, Denmark]) were used. The mirVana technology is single-colored and profiles 640 human, mouse, and rat miRs (including 154 Ambion miRs) using amine-modified DNA probes. The miRCURY microarray is dual-colored (to accommodate parallel hybridization of a reference sample) and contains LNA probes for 342 mouse and 146 Exiqon miRs. Note, that the Ambion and Exiqon company miRs are not entered into the Sanger miR Database [37]. P347S, 129 and c57 retinal samples were outsourced to Ambion Inc., and P347S retinal, c57 retinal, c57 brain and mouse platform samples were outsourced to Exiqon for miR profiling. All samples and replicates were analyzed on separate miR microarrays.

### mirVana miR microarray analysis

The mirVana miRNA Labeling Kit (Ambion Inc.) was used to label the samples with Cy5. The labeled samples were denatured and hybridized to the array for 12 to 16 hours at 42°C. Low stringency washes were followed by a high stringency wash to remove nonspecific binding to the array probes. The arrays were dried and images were acquired using the Axon^® ^GenePix 4000B scanner and GenePix software (Molecular Devices Ltd., Wokingham, UK). The raw signal for each probe was obtained by subtracting the maximum of the local background and negative control signals from the foreground signal. The data was pre-processed to remove poor-quality spots and normalization was used to remove any systematic bias. Global normalization of the microarrays was undertaken using the variance stabilization normalization [56] method. The resulting generalized log_2 _values were used in further data analysis.

### miRCURY LNA miR microarray analysis

Using the miRCURY™ LNA miR Array Labeling kit (Exiqon), experimental samples and a reference sample were labeled in separate reactions with Hy3 and Hy5, respectively. Labeled experimental and the reference sample were combined, denatured, and hybridized to microarrays at 65°C for 16 to 18 hours. Low stringency and high stringency washes were carried out and the microarrays dried. Images were acquired using the Axon^® ^GenePix 4000B scanner and GenePix software. The data was pre-processed and normalized using the global locally weighted scatterplot smoothing procedure [57]. Normalized log_2_-transformed Hy3/Hy5 ratios were used for further analysis.

### Data availability

Microarray data from the above studies are available at the public database Array Express [58] using the following accession numbers: E-TABM-329 (miRNA expression in diseased mouse retina) and E-TABM-332 (comparative miRNA profile of retina, brain, and RP).

### Quantitative real-time RT-PCR

Two-step qPCR was performed using ABI's TaqMan miR Assay (Applied Biosystems, Foster City, CA, USA), in accordance with the manufacturer's recommendations. Briefly, 10 ng total RNA was reverse transcribed with miR specific primers in 15 μl reaction volumes. Reverse transcription reactions were diluted 60-fold and 5 μl was amplified in triplicates by TaqMan qPCR on a 7300 Real Time PCR System (Applied Biosystems); quantification was performed utilizing the comparative Ct method [59]. RNU19 was employed as an internal control; log_2_-transformed miR/RNU19 expression ratios were used for further analysis.

### miR *in situ *hybridization and microscopy

5'-Digoxigenin (DIG) labeled, LNA-modified oligonucleotide ISH probes were purchased from Exiqon for the following mouse miRs: 1, 9*, 26b, 96, 129-3p, 133, 138, 181a, 182 and 335, and let-7d (including sense-159) as background control. Paraformaldehyde-fixed eyes were cryoprotected, cryosectioned (12 μm), thaw-mounted onto 3-aminopropyltriethoxysilane-coated microscope slides, and stored at -20°C. Sections were post-fixed in 4% paraformaldehyde and treated with diethyl-pyrocarbonate before a 2-hour pre-hybridization step in hybridization solution (50% formamide, 5 × sodium chloride/sodium citrate [SSC; pH 6.0], 0.1% Tween, 50 μg/ml heparin, and 500 mg/ml yeast tRNA). Sections were hybridized with LNA probes at 20 nmol/l concentration at the melting temperature (Tm) minus 21°C in a humidified chamber for 16 to 18 hours. Hybridized sections were then washed with 50% formamide and 2 × SSC at the hybridization temperature. Following 1 hour of blocking in 2% sheep serum, 2 mg/ml bovine serum albumin in phosphate-buffered saline (PBS) with 0.1% Tween, the slides were incubated with anti-DIG/alkaline phosphatase antibody/enzyme conjugate (1:2,000; Roche Diagnostics Ltd, Burgess Hill, UK) overnight at 4°C. Following successive washes in PBS with 0.1% Tween, the sections were incubated with nitroblue tetrazolium and 5-bromo-4-chloro-3-indoyl phosphate substrate (NBT-BCIP; Roche) for up to 48 hours. The reaction was stopped by washes in PBS, nuclei were counterstained with 4',6-diamidine-2-phenylindole-dihydrochloride. Sections were analyzed by bright field normal and phase-contrast as well as fluorescent microscopy using an Axiophot microscope (Carl Zeiss Ltd, Hertfordshire, UK). Corresponding images were overlaid in Adobe Photoshop (Adobe Systems Europe Ltd, Glasgow, UK).

### Bioinformatics

Potential retina specific targets of miR-1, miR-96, miR-133, and miR-183 were generated through computational means. Mouse transcripts predicted to be microRNA targets were retrieved from the Sanger microRNA Database [37]. Predictions were computed using microRNAanda version 3 [39] and filtered for *P *orthologous group value < 0.05. IDs of genes expressed in retina were downloaded from seven selected mouse eye libraries in UniGene (build #164) as follows: Lib.8659, NIH_MGC_94 (23,422 expressed sequence tags [ESTs] grouped into 7,638 UniGene entries); Lib.6780, NIH_BMAP_Ret4_S2 (19,072 ESTs grouped into 8,437 UniGene entries); Lib.5390, RIKEN full-length enriched, adult retina (6,089 ESTs grouped into 3,452 UniGene entries); Lib.15224, mouse retina, unamplified: mk/ml (4,658 ESTs grouped into 2,832 UniGene entries); Lib.20873, mouse retina, Y2H (nbk) (1,843 ESTs grouped into 1290 UniGene entries); Lib.12980, mouse adult retina (1,111 ESTs grouped into 872 UniGene entries); and Lib.6773, NIH_BMAP_Ret3 (961 ESTs grouped into 748 UniGene entries).

Additionally, genes were retrieved from two SAGE studies that determined genes expressed in the mouse retina and from an eye disease gene list at NEIBank [40] translated into mouse homologs: 3,516 UniGenes (with tag-level > 3) from Blackshaw and coworkers [41]; 3,475 UniGenes from Blackshaw and coworkers [42]; and mouse homologs of 488 eye disease genes from NEIBank [40].

Ensembl transcripts from microRNAanda prediction and UniGene entries from mouse retina libraries and lists were linked and extracted whenever gene names were available in both sources.

### Statistical analysis

Data from given sets were pooled and averaged, and standard deviation values calculated. Statistical significance of differences between datasets were determined using either Student's two-tailed *t*-test or analysis of variance; differences with *P *< 0.05 were considered statistically significant.

## Abbreviations

DIG, digoxigenin; EST, expressed sequence tag; INL, inner nuclear layer; ISH, *in situ *hybridization; LNA, locked nucleic acid; miR, microRNA; ONL, outer nuclear layer; P347S, pro347ser *RHO *transgene; PBS, phosphate-buffered saline; RP, retinitis pigmentosa; RT-PCR, reverse transcription polymerase chin reaction; qPCR, quantitative real-time RT-PCR; SAGE, serial analysis of gene expression; SSC, sodium chloride/sodium citrate.

## Authors' contributions

AP and GJF conceived and supervised the study. CJL, AP and GJF participated in study design and coordination. CJL and AP undertook the animal breeding, tissue collection, and RNA purification, microarray analysis, interpretation, qPCR validation, and ISH studies. ACI helped with the analysis of the Ambion microarray data. KH undertook the bioinformatics study, and CJL and AP interpreted the bioinformatics analysis. KH, AP, PFK, PH and CJL drafted the manuscript, and GJF approved the final manuscript. Financial support was provided by AP, PFK, PH and GJF. All authors read and approved the final manuscript.

## Additional data files

The following additional data are available with the online version of this paper. Additional data file 1 is a table listing retinal miR expression data from Exiqon microarray analysis. Additional data file 2 is a table listing retinal miR expression data from Ambion microarray analysis. Additional data file 3 is a table listing highly ranked retinal miR target genes predicted using miRanda.

The miRNA array data are available at Array Express [58] using accession numbers E-TABM-329 and E-TABM-332.

## Supplementary Material

Additional data file 1 Log_2 _median ratios are given for c57, 129, and P347S retinas at age 1 month; analysis was carried out in triplicate. *P *values by Student's *t*-test are also provided. Click here for file

Additional data file 2 Global normalised log_2 _ratios are given for c57, 129, and P347S retinas at age 1 month; analysis was carried out in triplicate. *P *values by analysis of variance and Student's *t*-test are also provided. Click here for file

Additional data file 3 Presented is a table listing highly ranked retinal miR target genes predicted using miRanda. Click here for file
